# Analysis of the IDS Gene in 38 Patients with Hunter Syndrome: The c.879G>A (p.Gln293Gln) Synonymous Variation in a Female Create Exonic Splicing

**DOI:** 10.1371/journal.pone.0022951

**Published:** 2011-08-04

**Authors:** Huiwen Zhang, Jing Li, Xinshun Zhang, Yu Wang, Wenjuan Qiu, Jun Ye, Lianshu Han, Xiaolan Gao, Xuefan Gu

**Affiliations:** Department of Pediatric Endocrinology and Genetic Metabolism, Xinhua Hospital, Shanghai Institute for Pediatric Research, Shanghai Jiao Tong University School of Medicine, Shanghai, China; University Hospital Vall d'Hebron, Spain

## Abstract

**Background:**

Hunter syndrome (mucopolysaccharidosis type II, MPS II) is a rare disease inherited in an X-linked autosomal recessive pattern. It is the prevailing form of the mucopolysaccharidoses in China. Here we investigated mutations of *IDS* (iduronate 2-sulfatase) gene in 38 unrelated Chinese patients, one of which is a female.

**Methods:**

Peripheral leucocytes were collected from the patients and the *IDS* gene was amplified to looking for the variations. For a female patient, the X chromosome status was analyzed by androgen receptor X-inactivation assay and the mutation impact on RNA level was further performed by reverse transcription polymerase chain reaction.

**Results:**

We discovered that point mutations constituted the major form while mutations in codon p.R468 defined the largest number of patients in our cohort. Consistent with data from other ethnic groups, exons 9 and 3 had comparatively more mutations, while exon 2 had quite a few mutations unique to Chinese patients. Of the 30 different mutations identified, only 9 were novel: one was a premature termination mutation, i.e., c.196C>T (p.Gln66X); three were missense mutations, i.e., c.200T>C (p.Leu67Pro), c.215T>C (p.Leu72Pro), c.389C>T (p.Thr130Ile); one was a small deletion, i.e., c.1104_1122del19 (p.Ser369ArgfsX16); and one was a deletion that spanned both exons 8 and 9 deletion leading to gross structural changes in the IDS gene. In addition, a synonymous mutation c.879G>A (p.Gln293Gln) was identified in a female Hunter disease patient, which resulted in loss of the original splicing site, activated a cryptic splicing site upstream, leading to a 28 bp deletion and a premature termination at p. Tyr285GlufsX47. Together with concurrent skewed X-inactivation this was believed to facilitate the development of Hunter disease in this girl.

**Conclusions:**

In conclusion, the molecular analysis of *IDS* gene in Chinese patients confirmed the Hunter disease diagnosis and expanded the mutation and clinical spectrum of this devastating disorder.

## Introduction

Hunter syndrome (MPS II; OMIM 309900), is caused by deficiency of the lysosomal enzyme iduronate 2-sulfatase (IDS, EC 3.1.6.13) which functions in the catabolism of two glycosaminoglycans, dermatan sulphate and heparan sulphate. A deficiency of IDS leads to dermatan sulphate and heparin sulphate accumulation mainly in connective tissue, liver, spleen, and brain, with excretion in the urine, even during fetal development. After birth, disease symptoms present at various times reflecting a wide disease spectrum, from a mild form without cognitive involvement to a severe form with early onset, mental retardation, rapid progression and death in the first or second decade of life.

The *IDS* gene located on chromosome Xq28 consists of 9 exons encoding a 550-amino acid IDS protein. The presence of several transcripts in normal humans reflects a complex system of intron splicing and strongly suggests that the *IDS* gene is susceptible to splicing mutations. About 80 kb downstream of the transcribed *IDS* gene, there is an *IDS* pseudogene, called *IDS2*. It is homologous in exons 2 and 3, and introns 2, 3, 7 of transcribed *IDS* [1], where exon 3 is 100% identical and the other sequences are 96% identical. The presence of a pseudogene likely renders the *IDS* gene more prone to recombination [2]. To date, more than 350 mutations have been identified in the *IDS* gene (www.hgmd.org), with exonic point mutations comprising half the mutations, followed sequentially by small deletions, altered splicing, gross deletions, small insertions, complex rearrangements, small indels, and gross insertions/duplications,.

As suggested previously [3] the IDS genotype correlates in general with the clinical phenotype. Usually, gross structural changes including large deletions and gene-pseudogene recombinations are associated with severe phenotypes, while small gene variations may result in severe to mild clinical presentations. As siblings from the same family may show completely different clinical courses [4], it is highly likely that in addition to the primary action of *IDS* gene, other factors modify its effects on the clinical presentation of Hunter syndrome.

MPS II is the predominant form of mucopolysaccharidoses among Japanese, Koreans, Jews [5], and Chinese [6], [7], which is different from other ethnic associations. For example, MPS III (where there are 4 distinct genetic entities) has a higher incidence than other forms of MPS in Western Australians [8], Germans [9], and Netherlanders [10], while Brazilians have a high incidence of MPS VI [11].

Herein we report the *IDS* mutation spectrum among the largest collection of Chinese patients with Hunter syndrome and compare it with those in other populations. Although this disorder affects primarily males, nearly a dozen female patients have been documented so far [12], [13] where most female patients arise from skewed X-inactivation. Here the underlying molecular mechanism for the disease in a Chinese female was also investigated.

## Materials and Methods

### Patients

Informed consents from the parents were obtained as approved by the local Institutional Review Ethics Board. A total of 38 unrelated patients with Hunter syndrome, one of which was female, were registered from our genetic metabolism clinic. These patients initially suspected of having mucopolysaccharidosis due to growth retardation, short stature, or bone dysmorphology examined by x-ray were confirmed by urine excretion of dermatin sulphate following glycosaminoglycan electrophoresis, and by low IDS activity in leukocytes from peripheral blood. A second sulphatase, i.e. arylsulfatase A, was measured to preclude the multiple sulphatase deficiency. Clinically patients were classified as severe if they had mental retardation and as mild based on normal mental status, as reported by Froissart et al [14]. Measurement of IDS activity in leukocytes was carried out in accordance with Voznyi et al [15]. The gender identity of the female patient with Hunter syndrome was confirmed by negative *SRY* (*sex determining region Y*) gene amplification.

### IDS Genomic DNA analysis

Genomic DNA was extracted from peripheral blood using the DNA isolation kit RelaxGene blood DNA DP319-01 (Tiangen, China) according to the manufacturer's protocol. All exons and their flanking regions of the IDS gene were amplified with primers designed by Primer3 except for exon 3, as reported previously [16]. Amplification reactions were performed in a total volume of 25 µL in the presence of 2.5 µL of 10× PCR buffer (Qiagen), 0.2 mmol/L of each dNTP, 0.4 Umol/L of each primers and 0.5 U of tag DNA polymerase (Qiagen), and 200 ng DNA. For amplification of Exon 1, 3, 5, 6, 7, 8 and 9, the samples were predenatured by heating to 95°C for 5 min, followed by 35 cycles consisting of denaturation at 94°C for 40 sec, annealing at 61°C for 40 sec, and extension at 72°C for 1 min, and subsequently for 10 min at 72°C. For amplification of exons 2 and 4, the conditions were the same as for other exons, except that the annealing temperatures were 60°C and 67.5°C, respectively. Amplicons were purified and sequenced directionally to search for mutations and/or variations using an ABI PRISM Dye Terminator Cycle Sequencing Kit, and an ABI 3700 automated fluorescent sequencer (PE Applied Biosystems, Foster City, CA).

The method, reported by Lualdi and colleagues [17], was used to search for gene-pseudogene recombinations in patients where no mutations were identified by exon sequencing, and in patients where any of the exons failed to be amplified. The first set of primers was also used to evaluate whether the pseudogene was involved in the large deletion of *IDS* gene. The negative amplification indicated the large deletion included both the *IDS* gene and the pseudogene.

### Androgen receptor X-inactivation assay

This assay was used to analyze the status of X chromosome inactivation in the female Hunter patient, which was performed as described [18]. The forward primer was FAM-labeled. PCRs were performed as documented with products run on an ABI Prism 3730 DNA Analyzer (Applied Biosystems) and analyzed using the Peak Scanner Version 1.0 software (Applied Biosystems).

### IDS cDNA analysis

Since a synonymous variation was identified in the female Hunter syndrome patient, the RNA of her and that from her sister was further analyzed to check the impact on RNA level. Total cellular RNA was isolated from peripheral blood using PAXgene™ blood RNA kit (Qiagen) with the protocol provided by the manufacturer and stored in the PAXgene™ blood RNA tube. RNA was then reverse transcribed using PrimeScript ™RT reagent kit (TaKaRa, Japan), with a reaction volume 10 µL containing 200 ng of total RNA, 1 µL of RT Enzyme Mix I, 2 µL of 5× Buffer, and 0.5 µL of Oligo dT Primer. The tube was incubated at 37°C for 15 min, then heated to 85°C for 5 sec to denature the PrimeScript® RTase. Primers were designed to cover the mutation found in the female patient (table 1), which was applied with 3 µL of reverse-transcribed product to amplify this 1242-bp sequence. The amplicons were first run on the 1% agarose gel to check whether the apparent molecular weights of amplicons were as expected and to evaluate any possible change of the IDS RNA in the patient. The amplicons were then sequenced bidirectionally.

**Table 1:** Primers used for amplifying exons and cDNA.

Name	Primer sequence ((5′→3′)	Annealing T(°C)	Length (bp)
e1F	CTGTGTTGCGCAGTCTTCAT	61	676
e1R	ATGCAGGAAAGGACAGATGG		
e2F	CCATCTGTCCTTTCCTGCAT	60	561
e2R	TAACAAGATGTCCCGCACAA		
e3F	GCTGTGGCGATGCTTACCTCTG	61	250
e3R	AAGAGAACCCAGACTCTGGACA		
e4F	GGCTTAGGGACCAGGAAGTC	67.5	508
e4R	AACAAGTAGCACCCACCAGC		
e5F	CCTGCCTGGAAAACAAGAAA	61	552
e5R	GGCCTTGACCTCTAAATCCC		
e6F	ACGTGGGGAATGCTAGTGAG	61	514
e6R	GTTGGGAGAGTCCTGATCCA		
e7F	GCTGTGACTCTGTGGGTGAA	61	627
e7R	CCAGGTTAAAAATGGGGGTT		
e8F	CAGCCTGTCAAGAATGAGCA	61	623
e8R	ACCCCCAAAGCCTATGATTC		
e9F	CATATGGAGCCCAGACAGGT	61	610
e9R	GGAAGGGAGCACATCACATT		
p-cDNA-F	GCGGCGGCTGCTAACTG	58	1242
p-cDNA-R	CAACTGTGAGGCGGAATCAA		

## Results

### Mutation spectrum of *IDS* in Chinese patients

Among these 38 unrelated Chinese patients with Hunter syndrome, 37 of them have been identified with *IDS* gene variations. All the variations were considered to be causative for Hunter disease, since they were not found in 100 alleles from normal people. No second variation existed in the same patient except in one male patient where two variations identified (see below). In one patient, no mutation was found after sequencing all coding exons and was found free of gene-pseudogene recombination.

A total of 30 different mutations, including 9 novel mutations and 21 previously reported mutations (Table 2), were identified. These were categorized as exonic point mutations, small deletions, intronic mutations, small insertions, gross deletions, and recombinations according their impact at the genomic level. While mutations were found in all exons they were concentrated in exon 9 (21%; 8/38), exon 2 (11%; 4/38) and exon 3 (11%; 4/38).

**Table 2:** Summary of mutations in a Chinese cohort of Hunter syndrome and their corresponding phenotypes.

Category	Mutation	Consequence	Occurrence	Position	Phenotype	Status
Exonic point mutation	c.1402C>T	p.Arg468Trp	3	ex.9	severe	reported
	c.1327C>T	p.Arg443X	2	ex.9	mild	reported
	c.1403 G>A	p.Arg468Gln	1	ex.9	severe	reported
	c.[1267C>T+1402C>T]	p.[Pro423Ser+Arg46Trp]	1	ex.9	severe	novel
	c.1505G>A	p.Trp502X	1	ex.9	severe	reported
	c.196C>T	p.Gln66X	1	ex.2	severe	novel
	c.200T>C	p.Leu67Pro	1	ex.2	severe	novel
	c.215T>C	p.Leu72Pro	1	ex.2	severe	novel
	c.238C>T	p.Gln80X	1	ex.2	severe	reported
	c.263G>A	p.Arg88His	2	ex.3	severe	reported
	c.323A>C	p.Tyr108Ser	1	ex.3	mild	reported
	c.389C>T	p.Thr130Ile	1	ex.3	mild	novel
	c.941T>C	p.Leu314Pro	1	ex.7	mild	reported
	c.998 C>T	p.Ser333Leu	1	ex.7	severe	reported
	c.514C>T	p.Arg172X	1	ex.5	mild	reported
	c.879G>A	p.Gln293Gln (p.Tyr285GlufsX47)	1	ex.6	mild	novel
	c.1122C>T	p.Gly374Gly (20 amino acid deletion)	1	ex.8	severe	reported
	c.95C>A	p.Ser32X	1	ex.1	severe	reported
Small deletion	c.1104_1122del19	p.Ser369ArgfsX16	1	ex.8	severe	novel
	c.596_599del4	p.Lys199AspfsX14	1	ex.5	severe	reported
Small insertion	c.161–162insA	p.Tyr54X	1	ex.2	severe	novel
Intronic mutation	c.880-8A>G		1	intron 6	mild	reported
Gross deletion	EX8_9del		2	ex.8 and ex.9	severe	novel
	EX8del		2	ex.8	severe	reported
	EX_9del (pseudogene intact)		1		severe	reported
	EX1_9del (including pseudogene)		1		severe	reported
	EX4del		1	ex.4	severe	reported
Recombination	type A		2		severe	reported
	type B		1		severe	reported
	type C		1		severe	reported

Exonic point mutations were predominant and comprised 58% (22/38) of all alleles. One synonymous mutation c.1122C>T (p.Gly374Gly), common in Caucasians [19], [20] that lead to a splice mutation with deletion of 20 amino acids of IDS protein [21], was included in this category for the sake of simplicity. Also included was a new same sense mutation c.879G>A (p.Gln293Gln) identified in a female with Hunter syndrome, proved to result in a splice mutation in our report. The second largest group of mutations involved large structural changes, including gross deletions and gene-pseudogene recombinations, comprised 29% (11/38) of the total.

Unlike most mutations that were unique or individualized, the codon 468 position could be considered a “hot” spot as 5 patients displayed mutations (13%, 5/38), with 4 of them p.Arg468Trp. Two other previously reported mutations, p.Arg443X and p.Arg88His, each occurred twice.

### Novel Mutations

**Figure 1:**
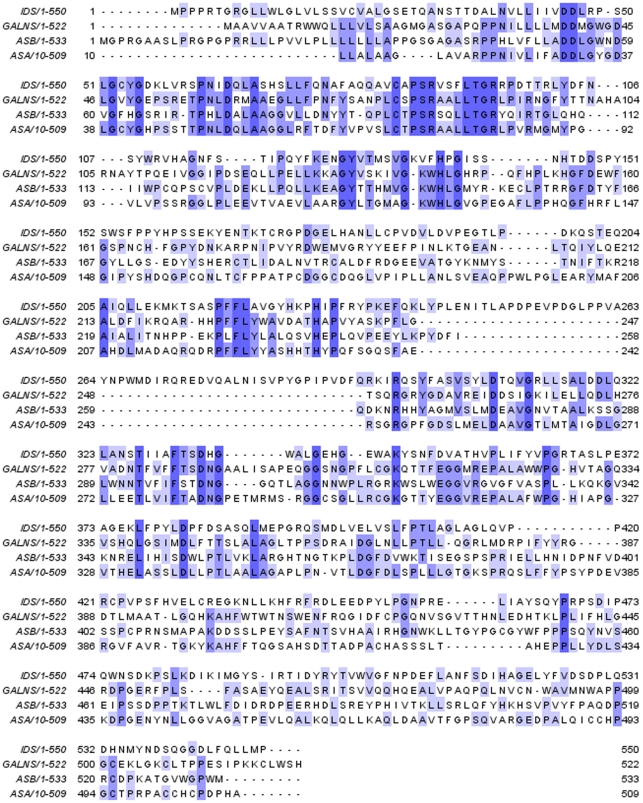
Homology alignment of amino acid sequences among IDS, N-acetylgalactosamine-6S sulfatase, arylsulfatase A, and arylsulfatase B. Residues identical in at least two of four sulfatases were shaded by blue, with more identity, the color used deeper. GALNS, ASA, and ASB were the short forms of N-acetylgalactosamine-6S sulfatase, arylsulfatase A, and arylsulfatase B, respectively.

Of the 22 exonic point mutations identified, most had been reported while in 6 patients with novel mutations, five of them were single changes, while one patient had two changes. The five single mutations were: c.196C>T (p.Gln66X), c.200T>C (p.Leu67Pro), c.215T>C (p.Leu72Pro), c.389C>T (p.Thr130Ile) and c.879G>A (p.Gln293Gln). In one patient and his mother, a novel variation c.1267C>T (p.Pro423Ser) was found to coexist with a previously reported mutations, c.1402C>T (p.Arg468Trp). The p.Leu67Pro, p.Leu72Pro, and p.Thr130Ile, all located near the active centre C84 of IDS, appear conserved, being identical in three of the four human arylsulfatases, including N-acetylgalactosamine-6S sulfatase, arylsulfatase A, and arylsulfatase B. However, Proline 423 is less conserved, being identical in two of the four arylsulfatases (Figure 1). Neither p.Leu67Pro nor p.Leu72Pro was likely to activate new cryptic splicing sites as judged from the Maxent program (http://genes.mit.edu/burgelab/maxent/Xmaxentscan_scoreseq.html).

Although only 3 individuals (8%, 3/38) had small deletion/insertion mutations, two of them were novel. The small deletion c.1104_1122del19 in exon 8 led to an amino acid change at codon 369 and a premature termination codon 16 amino acids later. The small insertion c.161–162insA generated an early premature stop codon at residue 54.

The gross structural changes seen have been identified in other ethnic groups, except for the deletion of both exon 8 and 9 of *IDS* gene which has not been documented previously. This mutation occurred in two patients in our cohort. With the deletion of these two exons, the cDNA was presumed to encode a peptide containing about 60% of the entire IDS protein. Whether the deletion extended to IDS pseudogene and gene W was not further investigated in these two patients. If a residual gene W were to exist, a fusion protein between partial IDS and gene W would be synthesized as reported [22].

### The molecular analysis of the female patient

The variation c.879G>A, located in the last nucleotide of exon 6, was identified in the female Hunter patient. Her mother and a female sibling were carriers of this mutation. The father did not carry any mutation at this site.

**Figure 2:**
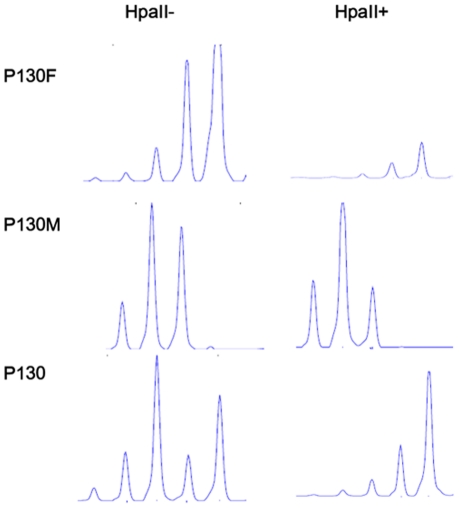
Androgen receptor X- inactivation assay in the female patient with Hunter syndrome. An X-inactivation pattern >95∶5 was noted in the female patient (P130), while her mother (P130M) showed an pattern 60∶40. After digestion by HpaII, the female patient showed the same signal as her father (P130F) indicating that the androgen receptor gene on the allele from her father was methylated and preserved.

Since only one mutation was identified in the female patient, and the gender authenticity was proved by negative SRY amplification, we investigated the methylation status of Androgen receptor to determine if skewed X-inactivation could be the basis for her disease. Her mother showed a 60∶40 X-inactivation pattern (Figure 2), which was a random form of this process and consistent with her carrier status. The pattern of X- inactivation in the female patient was >95∶5, which documents a skewed X-inactivation. She showed the same signal peak after HpaII digestion as her father indicating she received the androgen receptor allele from her father, i.e, the mutated IDS gene from the mother was selected for expression.

**Figure 3:**
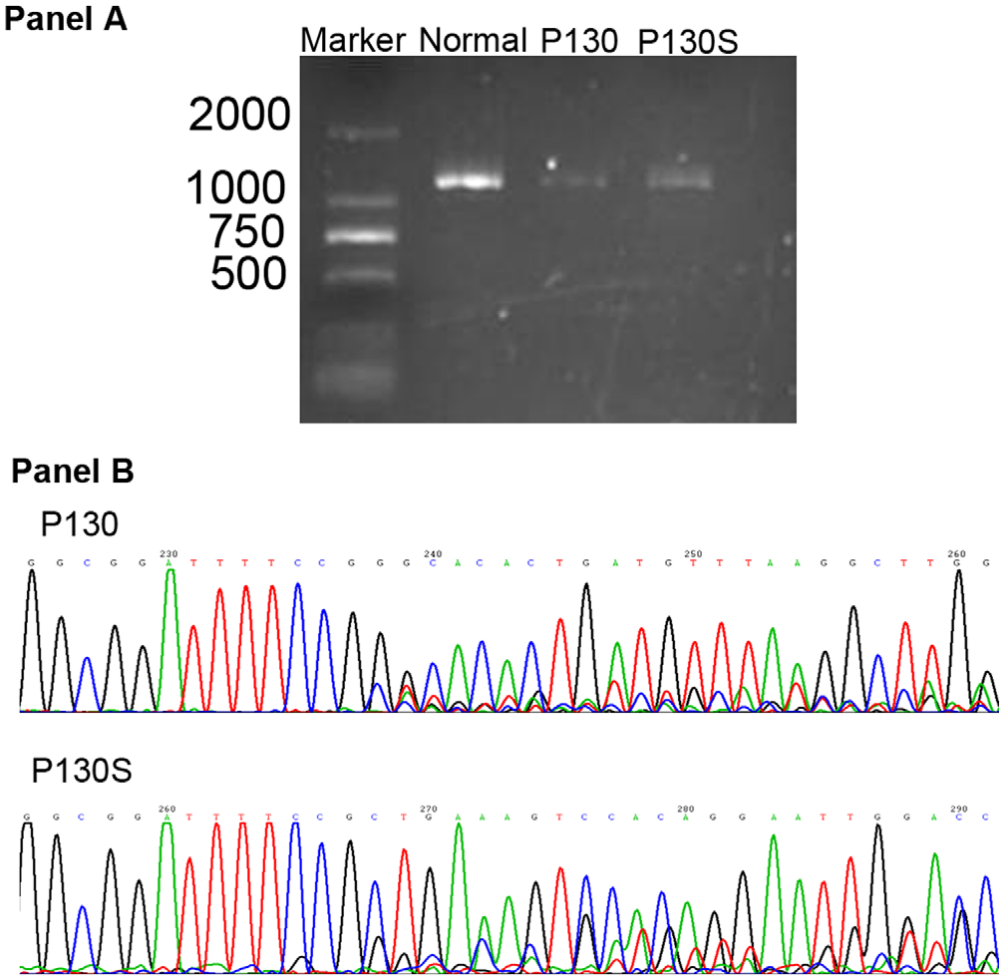
The molecular analysis at RNA level of *IDS* gene in a girl. Compared with normal female control, *IDS* gene presented remarkably low RNA expression in both the patient (P130) and her female sibling (P130S) (Panel A). The RNA sequencing revealed the patient (P130) had an abnormal sequence as the major transcript while her phenotypically normal sister had a normal sequence as the major transcript (Panel B).

ESE (exonic splicing enhancer) finder (http://rulai.cshl.edu/cgi-bin/tools/ESE3/esefinder.cgi) predicted that c.879G>A would disrupt the ESE site at end of exon 6 and lose the original 5′ splice donor site. To prove this prediction, RNA was isolated and amplified. We found the *IDS* RNA level (panel A of Figure 3) was greatly reduced in the female patients, as well as in her mutation-carrier sibling. Although no apparent difference of amplicons was shown on the agarose gel, their sequencing revealed the distinction between a patient and a mutation carrier. Since the mutation was located in the latter half of this amplicon, forward sequencing could not show the changed nucleotides (panel B of Figure 3 displays the reverse sequencing). It is notable that both the patient and the carrier had a major transcript, despite the existence of minor transcripts. By comparing the major transcripts with IDS cDNA reference (NM_000202.5), we could see that the carrier had a normal one, while the patient's is 28 bp shorter, which demonstrated an upstream alternative cryptic splice-site mutation was activated. These sequencing data were concordant with the respective enzymatic activities in their peripheral leucocytes, 1 and 8.71 nmol/4 h/mg (normal range: 16.4 to 83.2).

## Discussion

### Mutation category

The mutation spectrum in our series of unrelated 38 patients had the following features: i) exonic point mutations comprised the majority of all mutations; ii) the incidence of large structural changes was high, comparable to those found in France [23], Australia [24], Japan [25] ; iii) the small deletion/insertion mutation was quite rare (only 3 individuals belonged to this category) compared with Caucasian [19], [20], [26], and South Korean populations [27]; iv) the mutations involving introns were also scarce. Only one reported mutation c.880-8A>G had been identified in our group in contrast to data from a Portuguese study, where a high frequency (6/16) of intronic splicing mutation was identified [19].

### The most frequent mutation and mutation distribution

Arginine 468 is a recurrent point mutation in nearly all ethnic groups. In a group of 43 Japanese with Hunter syndrome, 5 had mutations in this codon. Three out of 5 had p.Arg468Gln, the other two had p.Arg468Trp and p.Arg468Leu, respectively [25]. In a report of United Kingdom patients, four individuals carried the p.Arg468Gln mutation [28]. The mutation p.Arg468Gln was also exclusive in 5 out of 40 Italian patients [29] whereas the p.Arg468Trp and p.Arg468Gln mutations occurred with equal frequency and comprised nearly half (6/14) of all mutations in Taiwanese [30].

In our group, R468 was mutated in 5 individuals, of whom three were p.Arg468Trp, one was p.Arg468Gln, and one showed the combination of p.Arg468Trp with p.Pro423Ser. The R468 mutation had been shown to coexist with p.Arg101Cys, a rare polymorphism [31]. The p.Pro423Ser has never previously been found in Hunter disease. Since this patient had a classical severe Hunter disease, and p.Arg468Trp was already known to be associated with a severe type by itself, p.Pro423Ser was predicted to make a minor contribution to the phenotype. It was likely that p.Pro423Ser was also a rare polymorphism and p.Arg468Trp was the pathogenic mutation for this patient. The p.Arg468Trp has comparatively higher occurrence in Chinese than in other populations.

The synonymous mutation c.1122C>T (p.Gly374Gly) leading to a 20 amino acid deletion of the IDS protein was the most common mutation in Spanish patients [20], and has a higher frequency in other Caucasian populations [24], [32], appeared randomly in our collection.

The IDS mutations widely distributed throughout the whole 9 exons were frequently located in exon 9, exon 8, and exon 3, as documented in Caucasians [21], [25] and Japanese patients [25]. Our data were largely in agreement except that p.Gln66X, p.Leu67Pro, and p.Leu72Pro were located in exon 2, which is distinctive and suggests exon 2 may be a unique “hot spot” in Chinese Hunter patients.

### Genotype-phenotype correlation

Consistent with previous reports, gross gene structure alterations were associated with a severe clinical phenotype, except for one patient with exon 4 deletion, who had an attenuated form, while Vafiadaki and co-workers [28] described a severe form with the same deletion.

Our patients with nucleotide 468 changes always had a severe phenotype, as for the Japanese [25], United kingdom [28], and Taiwanese groups [30], while in a few reports it was associated with mild clinical phenotypes [33].

As for the novel mutations, the nonsense mutation (p.Gln66X) would lead to deletion of about 90% of IDS amino acid sequence [34] and could explain a severe clinical presentation. Both p.Leu67Pro and p.Leu72Pro are conserved among human sulfatases, and located at an intermediate position of the first α-helix and β-sheet, respectively. Substitution by Proline confers exceptional conformation rigidity, would likely interrupt the secondary structure of IDS protein, and decrease the IDS activity significantly. Tyrosine at codon 130 is also conserved and conversion to the hydrophobic isoleucine would also lower the IDS activity.

### RNA splicing in female Hunter patient

Our female patient with Hunter syndrome inherited a single point synonymous *IDS* gene mutation, leading to abnormal mRNA splicing, from her mutation-carrying mother. Simultaneously she had skewed X-inactivaton of the paternal allele resulting in the maternal allele harboring the mutation being transcribed. This combination accounts for most of the female patients with an X-linked autosomal recessive disorder [18], [35]. She was expected to have expressed only the mutant cDNA. The existence of some minor transcripts besides the major mutated one support complexities in the regulation system of *IDS* gene transcription, which require further investigation, as Lualdi et al reported [36]. Beside the major normal transcript in her carrier sister, there was some small amount of abnormal transcripts. The larger parts of the abnormal transcripts were possibly rapid degraded after synthesis by nonsense-mediated mRNA decay since c.879G>A (p.Gln293Gln) introduced a premature termination codon at 47 amino acids later after the first amino acid change.

### Conclusion

Herein we report mutations in a large group of mainland Chinese patients with Hunter syndrome, who share similarities to those of other populations, while manifesting population unique features. The novel mutation expands the *IDS* gene mutation spectrum and contributes the knowledge about the IDS protein structure.
